# Vitamin D-rich marine Inuit diet and markers of inflammation – a population-based survey in Greenland

**DOI:** 10.1017/jns.2015.33

**Published:** 2015-12-16

**Authors:** L. K. Schæbel, E. C. Bonefeld-Jørgensen, P. Laurberg, H. Vestergaard, S. Andersen

**Affiliations:** 1 Department of Public Health, Centre for Arctic Health, Aarhus University, Aarhus, Denmark; 2 Department of Clinical Medicine, Arctic Health Research Centre, Aalborg University, Aalborg, Denmark; 3 Endocrine Research Unit, Department of Clinical Medicine, Aalborg University, Aalborg, Denmark; 4 The Novo Nordisk Foundation Center for Basic Metabolic Research, Section of Metabolic Genetics, Faculty of Health and Medical Sciences, University of Copenhagen, Copenhagen, Denmark; 5 Department of Geriatric and Internal Medicine, Aalborg University Hospital, Aalborg, Denmark; 6 Department of Internal Medicine, Queen Ingrid's Hospital, Nuuk, Greenland

**Keywords:** Inuit marine diet, Vitamin D, Inflammation, Arctic Greenland, Population-based studies, Dietary surveys and nutritional epidemiology, CRP, C-reactive protein, hsCRP, high-sensitivity CRP, POPs, persistent organic pollutants, YKL-40, chitinase-3-like protein 1

## Abstract

The traditional Inuit diet in Greenland consists mainly of fish and marine mammals, rich in vitamin D. Vitamin D has anti-inflammatory capacity but markers of inflammation have been found to be high in Inuit living on a marine diet. Yet, the effect of vitamin D on inflammation in Inuit remains unsettled. This led us to investigate the association between vitamin D and markers of inflammation in a population with a high intake of a marine diet. We studied 535 Inuit and non-Inuit living in West and East Greenland. Information concerning dietary habits was obtained by interview-based FFQ. Blood samples were drawn for analysis of 25-hydroxyvitamin D, high-sensitivity C-reactive protein (hsCRP) and chitinase-3-like protein 1(YKL-40). Participants were divided into three groups based on degree of intake of the traditional Inuit diet. The diet groups (Inuit diet/mixed diet/imported foods) were associated with vitamin D levels in serum (74·2, 69·8 and 52·9 nm; *P* < 0·001), hsCRP (1·6, 1·4 and 1·3 mg/l; *P* = 0·002) and YKL-40 (130, 95 and 61 ng/ml; *P* < 0·001), respectively. YKL-40 level decreased with rising vitamin D level in Inuit (Inuit diet *P* = 0·002; mixed diet *P* = 0·011). YKL-40 was lower in groups with higher vitamin D levels after adjusting for other factors known to influence inflammation (*P* < 0·001). This was not seen for hsCRP. In conclusion, vitamin D and markers of inflammation vary in parallel with the intake of the marine Inuit diet. Vitamin D levels were inversely associated with YKL-40 levels, but no association with hsCRP was found. The hypothesised anti-inflammatory effect of vitamin D was not supported. Other factors in the marine diet may be speculated to influence inflammation.

IHD and its complications are major causes of death^(^^1^^)^. The atherosclerotic process that leads to IHD has been investigated for decades, and it is now widely recognised that atherosclerotic lesions represent a series of cellular and molecular responses where inflammation plays an important role^(^^2^^)^.

The progression of atherosclerosis is linked to an imbalance of inflammatory and anti-inflammatory activities^(^^2^^)^. This can be estimated by measuring markers of inflammation in serum. Chitinase-3-like protein 1 (also known as YKL-40) is an emerging marker of inflammation secreted by macrophages in the human atherosclerotic vessel wall^(^^3^^)^. YKL-40 has been demonstrated to be an indicator of inflammation involved in the presence and progression of coronary artery disease^(^^4^^,^^5^^)^. C-reactive protein (CRP) is another marker of inflammation. It is an acute-phase reactant produced by hepatocytes stimulated by IL-6. CRP is known to reflect the inflammatory processes in the arteries^(^^2^^)^.

Vitamin D influences the inflammatory response by inhibiting the inflammatory process. This inhibition is mediated by down-regulating pro-inflammatory cytokines and up-regulating anti-inflammatory cytokines^(^^6^^)^. Vitamin D deficiency has been shown to be associated with inflammation^(^^7^^)^ as well as an increased risk of CVD and mortality^(^^8^^,^^9^^)^.

A low occurrence of IHD among pre-Western Inuit^(^^10^^)^ was linked to a diet with a high content of traditional Inuit food items that consisted mainly of free-living fish and marine mammals^(^^11^^)^. However, the occurrence of IHD among Inuit in Greenland has increased^(^^12^^)^ along with changes in diet and lifestyle^(^^13^^,^^14^^)^.

The traditional Inuit diet is a rich source of vitamin D and a decreased intake of traditional Inuit diet implies a decreased level of vitamin D in the population^(^^15^^)^. Furthermore, the Inuit diet associates with markers of inflammation^(^^16^^)^ and this association may be speculated to be influenced by vitamin D from the Inuit diet.

This led us to investigate the association between the intake of the traditional marine Greenlandic diet and vitamin D levels in plasma and two different markers of inflammation among Inuit and non-Inuit living in East and West Greenland with marked differences in dietary habits. We further assessed if vitamin D might influence markers of inflammation in addition to other factors known to affect inflammation.

## Subjects and methods

### Area of investigation

Greenland has 56 000 inhabitants, with approximately 1000 foreign citizens living and working in Greenland. Nuuk (64·15° N, 51·35° W) in West Greenland is the capital of Greenland with just under 16 000 inhabitants of whom 75 % are Inuit (Eskimo) and 25 % non-Inuit (Caucasians). Nuuk was established as a trading post under the Danish crown in 1728 and is now a modern city with access to a wide variety of food items, including take-aways that are supplementary to the traditional Greenlandic food items. In addition, a wide variety of imported food items is available in stores.

The Ammassalik district (65·35° N, 38·00° W) of East Greenland was isolated until 1884 and is still difficult to reach by sea because of pack ice from the northern icecap. It is sparsely populated; there are less than 3000 inhabitants (93 % Inuit) in an area of 243 000 km^2^. Tasiilaq is the main town of the Ammassalik district, which also includes five settlements. Tasiilaq has one store and five minor shops. The selection of imported food depends on sea ice and hence the opportunity for ships to call at the local harbour. Each of the settlements has one store with a limited selection that also depends on access by sea.

The capital city, town and settlements are all situated on fjords, which give the inhabitants access to the sea. This allows for both occupational and leisure time access to hunting and fishing.

### Subjects

Participants were Inuit (Greenlanders) and non-Inuit (all Caucasian Danes), men and women, aged 50 to 69 years. The investigation was performed in Nuuk, Tasiilaq, and the settlements Tiniteqilaaq, Sermiligaaq, Kulusuk and Kuummiut in the Ammassalik district^(^^13^^,^^15^^,^^16^^)^. Settlements with less than fifteen inhabitants in the selected age group were not included for practical reasons^(^^13^^)^.

In Nuuk, names and addresses were obtained from the hospital registration system. A random sample of 480 was selected (25 % of the total population aged 50 to 69 years).

In the Ammassalik district names and addresses were obtained from the National Civil Registration System in which every person living in Denmark, the Faeroe Islands and Greenland is recorded.

For all invited participants a letter of invitation was delivered by the local hospital porter or the nursing station attendant. Invitations were sent to non-responders three times. In all, 561 persons were invited and 535 (95 %) participated^(^^13^^)^.

In this study, an Inuit (Greenlander) was defined as a person born in Greenland with both parents born in Greenland as well.

This study was conducted according to the guidelines laid down in the Declaration of Helsinki and all procedures were approved by the Commission for Scientific Research in Greenland before the study was commenced (j. number 505-31). All subjects gave informed written consent, which was in Danish or Greenlandic by participant choice.

### Investigational procedures

The study was conducted at the local hospital or nursing station or as home visits if requested. One of the doctors in the study performed a physical examination, which included measurement of height without shoes, weight in indoor clothing and recording of major disabilities. The National Civil Registration System was used to obtain information about sex and age. A questionnaire in either Danish or Greenlandic was completed by interview performed by a Greenlandic interpreter or one of the investigating doctors. The interview-based questionnaires were used to obtain information about lifestyle patterns and dietary habits. The same interpreter was used at all sites of investigation.

A venous blood sample was drawn at the visit using minimal tourniquet. The blood sample was separated and stored at −20°C until analysis. Serum was missing in five participants.

### Dietary habits

The FFQ included traditional Greenlandic food items (seal, whale, wild fowl, fish, reindeer, musk ox, hare and lamb) and imported food items (pre-cooked meals, potatoes, vegetables, butter, cheese, eggs and fresh fruit). For each food item, the participants were asked to categorise how frequently they had an intake of the particular food item – ranging from never to daily^(^^13^^,^^15^^)^.

Each item was then given a frequency score calculated as the average number of d per month it was ingested^(^^17^^)^: daily intake = 30·4; 4–6 times/week = 21·7; 1–3 times/week = 8·7; 2–3 times/month = 2·5; 1 time/month = 1 and never = 0 d/month. Greenlandic food items were scored positively, imported food items were scored negatively. The sum of food frequency scores was calculated for all food items ingested by each participant. Participants were then categorised in three diet groups according to a scale on which a frequency score of 100 % represented a diet consisting of solely Inuit food items and a score of 0 % represented a diet consisting of solely imported food items. The three diet groups were composed as follows: diet group 1 had Inuit food frequency scores of >60 %; diet group 2 had scores of 40–60 %; and diet group 3 had scores <40 %.

### Assays

#### Plasma 25-hydroxyvitamin D

Plasma 25-hydroxyvitamin D_3_ and D_2_ were analysed by isotope dilution liquid chromatography–tandem mass spectrometry (LC-MS/MS) and calibrated to the National Institute of Standards and Technology standard^(^^18^^)^, as described in detail previously^(^^15^^)^.

#### High-sensitivity C-reactive protein

High-sensitivity CRP (hsCRP) levels were measured with a highly sensitive, latex particle-enhanced immunoturbidimetric assay (DAKO) with a measuring range of 0·2–80 mg/l and with a lower detection limit of 0·03 mg/l.

#### Chitinase-3-like protein 1

Plasma YKL-40 levels were measured with an ELISA method (Quidel). The measuring range of the assay was 20–300 ng/ml, with intra- and interassay CV of 5·8 and 6·0 %, respectively.

Serum from the participants was analysed in a random order with participant characteristics blinded to the laboratories for all assays.

### Statistics

Results are given as number of participants in groups, percentages, medians and quartiles. Groups were compared using the χ^2^ test and one-way ANOVA. The unpaired-samples *t* test was used for the pairwise comparison. The Kruskal–Wallis test was used for comparison of levels between several groups, and Kendall's tau rank correlation coefficient was used to describe a relationship between groups. YKL-40 and hsCRP were logarithmically transformed for the linear regression analysis using the natural logarithm for calculations. Dependent variables were YKL-40 and hsCRP; explanatory variables were age, BMI, sex, smoking, alcohol intake, diet, vitamin D and ethnicity.

MedStat software (Astra) was used for the random selection of participants in Nuuk. Data were processed and analysed using Stata version 13.1 software (StataCorp.). Two-sided *P* values less than 0·05 were considered significant.

## Results

The 535 men and women were aged 50–69 years. Clinical characteristics of the participants are shown in Table 1. The group of non-Inuit and mixed ethnicity (*n* 101) consisted of more men and they were slightly younger than the group of Inuit (*n* 434). More Inuit were smokers.

**Table 1.** Descriptives of participants in the study among populations in West and East Greenland living on traditional Inuit food and imported food items (Numbers of participants and percentages)

	Ethnicity						
	Inuit		Mix		Non-Inuit		
	*n*	%	*n*	%	*n*	%	P*
Age							
50–59 years	259	60	6	86	79	84	<0·001
60–69 years	175	40	1	14	15	16	
Sex							
Men	229	53	5	71	75	80	<0·001
Women	205	47	2	29	19	20	
Alcohol†							
0–7	274	64	5	72	51	55	0·45
8–14	95	22	1	14	24	26	
15+	57	14	1	14	18	19	
Smoking							
Never	56	13	1	14	25	27	0·002
Past	49	11	1	14	17	18	
Present	328	76	5	72	52	55	
Vitamin D supplements‡							
Intake	23	5	0	0	22	23	<0·001
No intake	411	95	7	100	72	77	
BMI							
<30 kg/m^2^	331	82	7	100	77	82	0·46
>30 kg/m^2^	73	18	0	0	17	18	

* Compared using the χ^2^ test.

† Units per week where one unit equals 8 g alcohol.

‡ Daily intake.

**Fig. 1.**
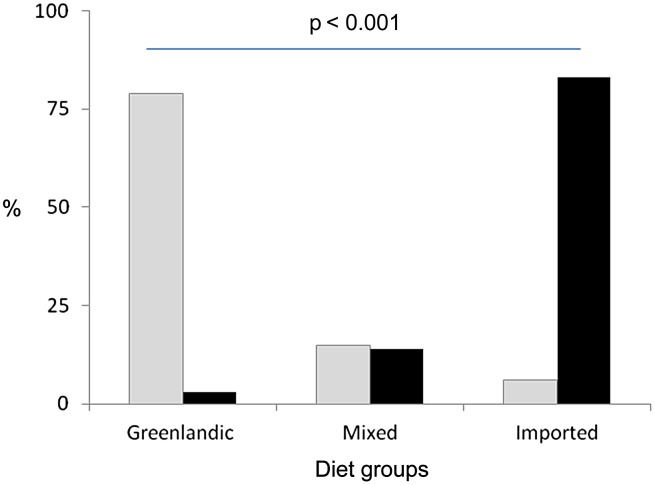
The distribution (%) of Inuit (░) and non-Inuit (■) in the three diet groups.

Fig. 1 illustrates the difference in intake of the traditional Greenlandic diet between the two ethnic groups who participated in the study. Markedly more Inuit than non-Inuit had a frequent intake of the traditional Greenlandic diet (79 %) while the reverse was the case for non-Inuit with 83 % living mainly on imported foods (*P* < 0·001).

The levels of vitamin D, YKL-40 and hsCRP for the three diet groups, mainly Greenlandic diet, mixed diet and mainly imported diet are shown in Table 2. Vitamin D as well as the inflammatory markers YKL-40 and hsCRP were markedly higher in the group living on the traditional Greenlandic diet when compared with the mixed and imported diet in the overall comparison (Table 2). In the pairwise comparison, vitamin D differed markedly between the three individual diet groups (Table 2). Findings were similar for YKL-40. The difference in hsCRP between the diet groups was marked only when comparing those with a high intake of the traditional Greenlandic diet and those with a high intake of imported food items while not when the group with the mixed diet was included in the comparisons (Table 2).

**Table 2.** Vitamin D and markers of inflammation in populations in Greenland divided into three diet groups (Medians and interquartile ranges (IQR))

	Diet group									
	Greenlandic		Mixed		Imported					
	Median	IQR	Median	IQR	Median	IQR	P*	*P*†	*P*‡	*P*§
Vitamin D (nm)	74·2	59·8–93·8	69·8	53·7–84·1	52·9	33·7–66·8	<0·001	0·014	<0·001	<0·001
YKL-40|| (ng/ml)	130·1	79·8–223·4	95·3	64·0–195·2	60·8	46·1–122·4	<0·001	0·021	<0·001	<0·001
hsCRP|| (mg/l)	1·6	1·1–4·0	1·4	0·7–3·5	1·3	0·5–2·3	0·002	NS	<0·001	NS

YKL-40, chitinase-3-like protein 1; hsCRP, high-sensitivity C-reactive protein.

* Compared using one-way ANOVA.

† *t* test, Greenlandic food *v.* mixed food.

‡ *t* test, Greenlandic food *v.* imported food.

§ *t* test, mixed food *v.* imported food.

|| ln-transformed for analysis.

Table 3 shows the influence of a number of factors on the two markers of inflammation. YKL-40 was influenced by age, BMI, smoking, diet and ethnicity but not by vitamin D in the crude comparisons. The adjusted comparisons added an influence of vitamin D and alcohol on YKL-40 with slightly decreased YKL-40 level with rising vitamin D. Interestingly, hsCRP was not influenced by vitamin D in the crude or adjusted comparison.

**Table 3.** Linear regression of factors important to markers of inflammation among populations in Greenland* (*β* Coefficients and 95 % confidence intervals)

		Univariate						Multivariate					
		YKL-40			hsCRP			YKL-40			hsCRP		
	*n*	*β*	95 % CI	*P*	*β*	95 % CI	*P*	*β*	95 % CI	*P*	*β*	95 % CI	*P*
Age	535	0·04	0·02, 0·05	<0·001	0·04	0·02, 0·05	<0·001	0·03	0·02, 0·05	<0·001	0·01	−0·00, 0·03	NS
BMI	505	−0·02	−0·04, −0·01	0·002	0·01	−0·01, 0·03	NS	−0·02	−0·03, −0·00	0·013	0·01	−0·01, 0·03	NS
Sex	535	0·09	−0·06, 0·23	NS	0·02	−0·18, 0·22	NS	0·00	−0·14, 0·15	NS	−0·12	−0·33, 0·09	NS
Smoking†	534	0·07	0·01, 0·13	0·032	−0·02	−0·10, 0·07	NS	0·04	−0·02, 0·11	NS	0·01	−0·08, 0·11	NS
Diet‡	535	−0·31	−0·40, −0·22	<0·001	−0·22	−0·34, −0·10	0·001	−0·16	−0·28, −0·03	0·013	−0·21	−0·33, −0·09	0·001
Vitamin D	529	−0·00	−0·00, 0·00	NS	0·00	−0·00, 0·01	NS	−0·01	−0·01, −0·00	<0·001	−0·00	−0·01, 0·00	NS
Alcohol§	526	0·04	−0·02, 0·11	NS	−0·16	−0·25, −0·08	<0·001	0·11	0·05, 0·17	<0·001	−0·14	−0·22, −0·05	0·002
Ethnicity||	535	−0·66	−0·83, −0·48	<0·001	−0·48	−0·73, −0·22	<0·001	−0·56	−0·82, −0·31	<0·001	−0·22	−0·60, 0·17	NS

YKL-40, chitinase-3-like protein 1; hsCRP, high-sensitivity C-reactive protein; NS, *P* > 0·10.

* Dependent variables were YKL-40 and hsCRP, both ln-transformed for analysis. Explanatory variables were age, BMI, sex, smoking, alcohol intake, diet, vitamin D and ethnicity.

† Cigarettes per d.

‡ Diet by three groups: Greenlandic, mixed, imported (Greenlandic diet reference).

§ Units per week.

|| Inuit *v.* non-Inuit (Inuit reference).

Ethnicity influenced YKL-40 and hsCRP, as both were clearly higher in Inuit than in non-Inuit both in the crude and adjusted comparisons (Table 3). Also, the traditional Inuit diet influenced YKL-40 and hsCRP with the highest levels among participants living on the traditional Inuit diet in both the crude as well as the adjusted analysis. The findings were similar for age with higher levels of both markers of inflammation with higher age though the influence was less pronounced.

**Fig. 2.**
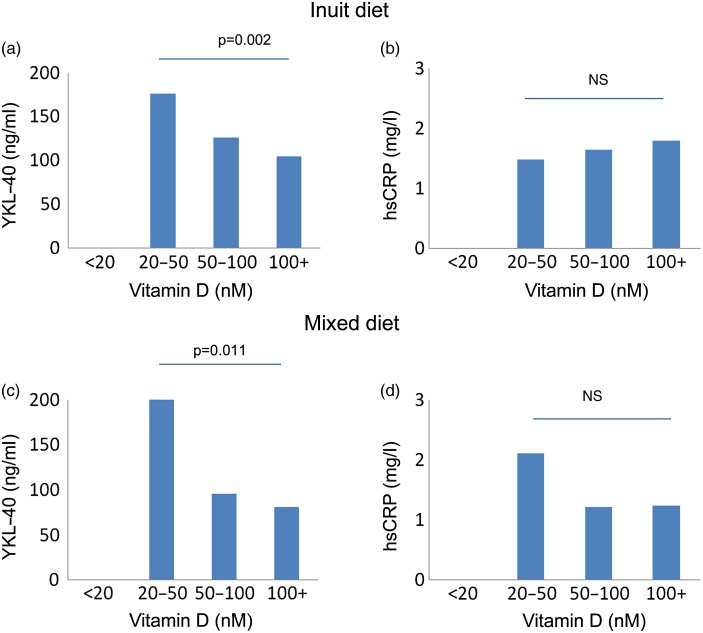
Vitamin D and markers of inflammation in serum from Inuit in the two diet groups with an intake of mainly Inuit diet and a mixed diet. Very few Inuit lived on imported food items (number of participants were 0, 4, 17 and 3 in the vitamin D groups <20, 20–50, 50–100 and 100+ nm). YKL-40, chitinase-3-like protein 1; hsCRP, high-sensitivity C-reactive protein.

The association between vitamin D on the two markers of inflammation is further detailed in Fig. 2, which shows YKL-40 (Fig. 2(a) and (c)) and hsCRP (Fig. 2(b) and (d)) by two diet groups and for Inuit participants only. It illustrates that YKL-40 levels differed between vitamin D-level groups for the Inuit participants living on the traditional Inuit diet and participants living on the mixed diet but not for those living on imported foods (data not shown). There was a distinct decrease in YKL-40 levels with rising vitamin D levels for Inuit living on the traditional Greenlandic diet (Kendall's tau, *P* = 0·002) and for those living on the mixed diet (*P* = 0·046). There was no difference in hsCRP with vitamin D level for either of the diet groups (Fig. 2).

## Discussion

This is the first population-based survey to study both vitamin D and markers of inflammation in relation to the traditional marine diet among Inuit and non-Inuit in Greenland. The main findings were markedly higher levels of the inflammatory markers YKL-40 and hsCRP as well as levels of vitamin D among participants with the highest intake of the traditional Inuit diet. Interestingly, we found only a limited association between vitamin D and markers of inflammation.

Inflammation is the net result of both anti- and pro-inflammatory factors. Increased inflammation has been shown in Inuit with a high intake of the traditional marine diet^(^^16^^)^ and with a high alcohol intake^(^^19^^)^ although the latter did not include dietary habits in their report. We hypothesised that vitamin D from the marine diet had an inflammation-dampening effect.

In the present report we measured two different markers of inflammation to portray the sum of inflammation. However, our data did not fully support our hypothesis. Thus, it may be speculated that the traditional Greenlandic diet may contain both anti- and pro-inflammatory components and that the latter dominates the inflammatory balance.

Sources of vitamin D in the Arctic include both dietary and dermal. Dermal vitamin D synthesis was hypothesised to be low both due to limited intensity of UVB light and due to low sun exposure of the skin as the general temperature discourages sunbathing, even during summer months. However, sun exposure does influence vitamin D levels at high latitudes as demonstrated in a study of Inuit and non-Inuit in North Greenland^(^^20^^)^. In that study Andersen *et al.* found indication of dermal vitamin D production in addition to the diet as a source of vitamin D. Still, the dominant contributor to vitamin D in populations in Greenland is the diet. Thus, previous studies support our finding of higher vitamin D levels among subjects with a high intake of Greenlandic food items. Rejnmark *et al*.^(^^21^^)^ surveyed Inuit living in the capital city Nuuk and Inuit and Danes living in Denmark. They reported higher vitamin D levels among participants with an intake of traditional Greenlandic food items compared with a Westernised diet. The influence of the traditional Inuit marine diet on vitamin D was detailed in a population-based study of Inuit and non-Inuit living in West and East Greenland^(^^15^^)^. The intake of the traditional marine diet contributed markedly to the vitamin D levels. Specifically, seal and whale were major contributors to vitamin D. The influence of the traditional Inuit marine diet on vitamin D has also been demonstrated in a recent study by Nielsen *et al*.^(^^22^^)^. They report a decrease in vitamin D levels over time along with decreasing intake of traditional Inuit food items. Hence, the influence of the traditional Inuit marine diet on vitamin D was documented in our study as well as in previous studies of Inuit in Greenland.

Vitamin D is speculated to have anti-inflammatory capacities. A correlation between vitamin D insufficiency and inflammatory diseases have been reported in a number of studies. These studies included patients with rheumatoid arthritis^(^^23^^)^, type 2 diabetes mellitus^(^^24^^)^, inflammatory bowel disease^(^^25^^)^, CVD^(^^26^^)^, acute infections^(^^27^^)^, and healthy older adults^(^^7^^,^^28^^)^. Hence, a potential anti-inflammatory role of vitamin D is extensively documented in observational studies. However, this association has been difficult to confirm in randomised controlled studies. Some have found an effect of vitamin D on inflammation^(^^29^^,^^30^^)^ while others have not^(^^31^^–^^34^^)^. The explanation for this discrepancy remains to be settled. The hypothesised inflammation-dampening effect of vitamin D was limited in our study, and also in the adjusted analysis. It could be speculated whether this anti-inflammatory effect is dimmed by other factors.

The traditional Greenlandic diet is mainly of marine origin and is dominated by fish and marine mammals^(^^11^^)^. The diet provides a range of nutrients to the population including vitamins A and D, Fe, I, P, Se and *n*-3 fatty acids^(^^13^^,^^35^^,^^36^^)^. However, the marine mammals and fish contain high levels of persistent organic pollutants (POPs)^(^^35^^,^^37^^,^^38^^)^. This conflict with a diet that contributes both important nutrients as well as harmful substances has been labelled ‘the Arctic dilemma‘^(^^35^^,^^37^^)^. POPs have an array of known effects in humans, including reduction of humeral immune response^(^^39^^)^ with increased inflammation^(^^40^^)^. Thus, POPs have been shown to induce oxidative stress *in vitro* in endothelial cells^(^^41^^)^, *in vivo* in rats^(^^42^^)^ and Kim *et al*.^(^^40^^)^ found increased inflammation in a study of POPs and inflammation in non-diabetic adults. It may thus be hypothesised that a pro-inflammatory effect of POPs explains the limited anti-inflammatory effect of vitamin D from the traditional Greenlandic marine diet in our study.

We used FFQ to assess the intake of the traditional Inuit diet and imported food items. FFQ do have limitations. However, FFQ is considered a valid method for assessing the intake of nutrients^(^^43^^)^. The FFQ used in the present study was interview-based and the questions were targeted well-known and clearly defined Greenlandic food items. Furthermore, it was validated in this population using cross-check questionnaires and iodine as a biomarker^(^^13^^)^. This clearly supports our use of the FFQ method. More non-Inuit than Inuit used supplements but the number of users was limited and supplement use did not corrupt our results. The population invited was limited in size. However, the total population of all of Greenland is only about 56 000 and the participation rate in our study was 95 %. The groups selected represent the diversity in dietary habits in all of Greenland with participants from the capital city as well as participants from a medium-sized town and from the most remote settlements in Greenland. Our study was strengthened by the dual approach to describe inflammation with two different inflammatory markers. YKL-40 is produced in the endothelial cells while hsCRP is produced in the hepatocytes^(^^2^^)^. Hence, the inflammatory process is documented by two different approaches. Residual confounding cannot be ruled out. However, we have re-run the regression analysis with only age and ethnicity in addition to vitamin D as explanatory variables and the two markers of inflammation as dependent variables. The results of these analyses were similar with a slight variation in coefficients but similar slope and significance levels. Thus, any residual confounding is likely to have limited influence on the results and hence not alter the conclusions.

In conclusion, the traditional Inuit diet contains important nutrients such as vitamin D and *n*-3 fatty acids that may act in an anti-inflammatory fashion. This favours the intake of the traditional marine diet. Still, based on our findings it may be hypothesised that pro-inflammatory factors are present in the marine diet. This needs to be elucidated to inform the discussion on ‘the Arctic dilemma’: to eat or not to eat traditional marine food items.
